# Mother-Child Dyadic Emotional Responses in Everyday Life: Moderation by Maternal Depressive Symptoms and Child Negative Emotionality

**DOI:** 10.1007/s10802-026-01462-x

**Published:** 2026-04-28

**Authors:** Xin Feng, Qingqing Yang, Yihui Gong

**Affiliations:** 1 Department of Human Sciences, College of Education and Human Ecology, The Ohio State University, 221B Ramseyer Hall 29 W Woodruff Ave., Columbus, OH 43210 USA; 2 Institute for Social and Health Equity, University at Albany, SUNY, Albany, NY USA; 3 Department of Health Policy, Management, and Behavior, University at Albany, SUNY, Albany, NY USA

**Keywords:** Mother-child emotional responses, Emotion coaching, Emotion dismissing, Maternal depression, Ecological momentary assessment (EMA)

## Abstract

Although emotion socialization during preschool years is well studied, how mother-child mutual emotional responses unfold in naturalistic contexts remains largely unknown. This pilot study examined within-dyad associations between children’s emotion expressions and maternal socialization behaviors, and the moderating effects of maternal depressive symptoms and child negative emotionality. Forty mother-child dyads participated (42.5% girls; M_age_ = 5.16 years). Across two consecutive weekend days, mothers completed ecological momentary assessments 12 times, reporting children’s positive and negative emotion expressions, the intensity of child peak negative expressions, and their own emotion coaching (EC) and emotion dismissing (ED) behaviors. Children’s peak negative emotion was associated with mothers’ use of EC and ED, and the peak negative emotion and EC link was moderated by child negative emotionality. Maternal EC/ED also predicted children’s subsequent positive and/or negative emotion expressions, and the association between maternal ED and child negative expressions was further moderated by maternal depressive symptoms or child negative emotionality. Using an ecologically valid, real-time assessment approach, this study explored how the mother-child emotion socialization process unfolds in everyday life. Findings highlight the negative impact of maternal ED on children’s emotional experiences and highlight the potential value of early interventions targeting parental ED behaviors.

**Supplementary Information:**

The online version contains supplementary material available at 10.1007/s10802-026-01462-x.

Emotion socialization is central to children’s learning to express and regulate emotions in social interactions. This process unfolds as children observe parents’ behaviors and receive contingent parental responses to their emotion expressions (Morris et al., 2007). Increasingly, emotion socialization is conceptualized as dynamic, bidirectional interactional processes (M. H. Chan et al., 2022; Cole et al., 2017). However, how these processes operate in everyday life to shape children’s emotional outcomes remains unclear. Early emotion socialization exerts an enduring impact on a broad array of developmental outcomes extending well beyond childhood, as dysregulated negative emotion is implicated in multiple forms of childhood problems and later psychopathology (Goodman, 2007). For example, persistent irritability is a central feature of oppositional defiant disorder (ODD), a common early-onset disruptive behavior disorder, and maladaptive parent–child emotional processes have been shown to contribute to its development and maintenance (Zachary & Jones, 2019). Thus, understanding emotion socialization processes in naturalistic contexts represents a critical step toward addressing this knowledge gap and informing practices to improve positive developmental and health outcomes. Accordingly, the present study examined the prospective, temporal association between maternal emotion socialization behaviors and children’s emotion expressions to elucidate the dynamics of emotion socialization in naturalistic home contexts.

Preschool years are a developmental period marked by the transition from parent-supported co-regulation to independent self-regulation of emotions (Calkins, 2007; Feng et al., 2017), and the critical role of parental emotion socialization during this period has been well established. In this study, we focused on two dimensions of maternal emotion socialization behaviors: *emotion coaching* (EC) and *emotion dismissing* (ED). EC is characterized by parental acceptance of children’s negative emotions and parental support for problem-solving and acquiring emotional knowledge, and has been regarded as a positive parenting practice. In contrast, ED, characterized by parental neglect, minimization, and invalidation of children’s emotional experiences, has been considered negative. Originally conceptualized within the framework of meta-emotion philosophy (Gottman et al., 1996), these constructs have more recently been examined as emotion socialization behaviors in parent-child dialogue (Buckholdt et al., 2016; Dunsmore et al., 2016; Feng et al., 2025) and parent-child interaction (Gerhardt et al., 2020; Loop & Roskam, 2016).

Prior research has generally linked maternal EC with children’s better emotion regulation abilities (Ellis et al., 2014; Lunkenheimer et al., 2007), greater positive emotion during play interactions (Loop & Roskam, 2016), and fewer negative expressions (Wu et al., 2022). Randomized controlled trials of an EC-based intervention, such as Tuning into Kids, further demonstrated that promoting parental EC reduces children’s negative emotions and behavioral problems (R. F.-Y. Chan et al., 2021; Havighurst et al., 2010). Parental EC responses are particularly important to facilitate children’s emotional learning and foster intimacy in the parent–child relationship (Lunkenheimer et al., 2007). Findings on maternal ED, however, have been less consistent. Some studies have linked dismissive responses to poorer emotion regulation and greater emotional difficulties in children (Yap et al., 2008; Yi et al., 2016), whereas others have reported minimal effects in family interactions (Lunkenheimer et al., 2007).

While substantial evidence supports the role of maternal emotion socialization in shaping children’s emotional outcomes, critical gaps remain. In particular, relatively little is known about how the emotion socialization processes unfold in everyday, routine mother-child interactions (Sperling & Repetti, 2018). Furthermore, empirical studies examining the reciprocal influences between children’s emotions and maternal socialization behaviors are limited.

## Mother–Child Emotion Socialization as a Bidirectional Process in Everyday Life

Most research on young children’s emotion socialization has been conducted in laboratory settings. Laboratory observations provide controlled emotion-eliciting challenges that maximize standardization and comparability across individuals (Repetti et al., 2015) and have yielded valuable insights into parent–child emotion socialization. However, these brief and highly structured interactions often fail to reflect the complexities of real-life family interactions (Moskowitz et al., 2009), where children’s activities, social partners, and emotional contexts are fluid, and multiple social interactions can coincide (Bai et al., 2016).

To address this gap, the present study employed the ecological momentary assessment (EMA) to capture mother-child mutual emotional responses in the home environment. EMA involves momentary, repeated sampling of individuals’ behavior and experience in real time, which enables the examination of micro-level interpersonal interactions as they unfold in naturalistic settings (Shiffman et al., 2008). This approach reveals dynamic processes in mother-child emotion socialization that are difficult to observe in controlled laboratory conditions (Teti & Cole, 2011), thereby enhancing the ecological validity of research. Recent studies demonstrate EMA’s utility in documenting daily parent-adolescent emotion coregulation within families. For example, Griffith and Hankin (2021) found that adolescents’ high-arousal positive affect (e.g., excited, energetic) increased after communicating their emotions to parents, and parents’ positive affect increased following their validating responses.

Despite these advantages, EMA methods have been rarely applied to preschool-age children, who typically cannot self-report their momentary experiences (Heron et al., 2017; Wen et al., 2017). Moreover, most EMA studies of parent-child interaction have focused on broad parenting constructs, such as parental support or relationship quality, and adolescent emotional outcomes (Allen et al., 2016; Bi et al., 2024; Janssen et al., 2021). Far less is known about the dynamics of naturalistic emotion socialization in early childhood, when both emotional expression and regulatory behaviors shift rapidly (Cole et al., 2017; Dennis-Tiwary, 2019). Capturing this process in daily life requires examining the temporal coupling between parents’ behaviors (e.g., EC and ED) and children’s emotional expressions, specifically, whether maternal regulation efforts modify children’s ongoing emotional states and, conversely, whether children’s emotions elicit parental responses (Cole et al., 2017). The present study investigated these temporal, bidirectional relations in everyday mother-child interactions.

## Maternal and Child Characteristics Influencing Emotion Socialization

Parents naturally adapt their behaviors in response to children’s emotional state to support their emotional development (Laukkanen et al., 2014). However, the effectiveness of parenting behaviors is shaped by parental and child characteristics. Belsky’s family process model posits that parenting is influenced by multiple attributes of the parent, child, and family context. In particular, this model highlights parental psychological functioning (e.g., depression) and children’s negative emotionality as central determinants of parenting quality (Belsky, 1984).

Extensive research links maternal depression, both clinical and subclinical, with disruptions in maternal affect, cognition, and behavior that compromise parenting quality (Goodman, 2007). Mothers experiencing depression often struggle to flexibly adjust their responses to children’s cues to provide emotional support (Goodman & Gotlib, 1999). Maternal depression also undermines the quality of mother–child interactions (Hummel et al., 2016) and alters emotion socialization strategies (Bao & Kato, 2020). For example, mutual displays of positive emotion in mother–toddler dyads were only observed in mothers with low depressive symptoms. Similarly, preschoolers exposed to both maternal depression and maladaptive emotion socialization showed elevated negative emotion in lab assessments (Hooper et al., 2015). An EMA study further documented that adolescents of parents with depression were at elevated risk for experiencing depressed affect (Sarigiani et al., 2003). Together, these findings suggest a moderating effect of maternal depression on emotion socialization processes.

Children’s negative emotionality, temperamental tendency to experience negative emotions, may further shape parenting dynamics. Although momentary negative emotion expressions capture children’s situational responses in specific contexts, negative emotionality reflects a relatively stable disposition that characterizes the frequency and intensity with which children experience negative emotions across contexts and over time. Thus, negative emotionality may shape how children respond to parental regulatory efforts. Moreover, from a transactional perspective of development (e.g., Sameroff, 2010), temperamental emotionality shapes the emotional climate of parent-child interactions and influences how parents respond to children’s emotion expressions (Laible & Panfile, 2009). Consistent with this, maternal support has been found to more effectively reduce children’s negative emotions among those lower in negative emotionality (Wu et al., 2017). These findings suggest the need to consider both parent and child vulnerabilities in understanding the emotion socialization processes.

## The Present Study

In this pilot study, we aimed to provide a nuanced depiction of the emotion socialization process in naturalistic settings by employing an EMA framework that simultaneously captured children’s emotional states and maternal regulatory behaviors within the flow of daily life. This study also responded to recent calls for integrating daily-level parent and child emotional regulation in dyads (Gadassi-Polack et al., 2025). As maternal depression is a robust risk factor for both maladaptive parenting and children’s dysregulated emotions (Feng et al., 2008; Goodman, 2007; Hooper et al., 2015), and children’s negative emotionality sets the tone for their emotion expression, we further explored how these characteristics shaped mother-child mutual emotional responses. We hypothesized that (1) child peak negative expression would be associated with decreased maternal EC and increased ED behaviors; (2) maternal EC would predict children’s decreased negative and increased positive expressions, whereas ED would predict increased negative and decreased positive expressions; and (3) maternal depressive symptoms and child negative emotionality would moderate the prospective within-dyad association (a) between child peak negative expression and maternal EC/ED responses, and (b) between maternal EC/ED and children’s subsequent positive/negative expressions.

## Method

### Participants

Participants were drawn from a larger study examining maternal depression and children’s emotion regulation. Dyads were recruited in a Midwestern city through preschools and daycare centers, primary care clinics, and social media advertisements. In the larger study, maternal eligibility was assessed using the *Structured Clinical Interview for DSM–5 Disorders* (SCID-5; First et al., 2015); children were screened with the *Wechsler Preschool and Primary Scale of Intelligence – Fourth Edition* (WPPSI–IV; Wechsler, 2012) and maternal reports on the *Pervasive Developmental Disorders Screening Test–II* (PDDST–II; Siegel, 2021). Inclusion criteria for mothers were: (1) age 21 years or older, (2) having a biological child aged 3.5–4 years, and (3) having no history of psychotic symptoms or bipolar disorder, and no substance use disorder in the past six months. Children were 3.5–4 years old, free of developmental disorders or delays, and had an IQ above 70. Only one child per family participated.

The present study was a follow-up of the larger project, and the current sample consisted of 40 mother-child dyads, of which 40.0% of the mothers (*n* = 16) had a history of MDD during the child’s lifetime. Children (17 girls, 42.5%) were, on average, 5.16 years old (SD = 0.29) at the time of EMA assessment. Most mothers identified as White (92.5%; *n* = 37), were married (90.0%; *n* = 36), and held at least a bachelor’s degree (85.0%; *n* = 34). The mean family income-to-need ratio (i.e., household income divided by 100% of the federal poverty line, adjusted for household size) was 3.93 (*SD* = 1.64), reflecting a middle-class sample (Conger et al., 1997).

### Procedures and Measures

Children’s positive and negative emotion expressions and mothers’ EC and ED behaviors were assessed in naturalistic home settings using the EMA via a smartphone app (MovisensXS, Movisens Inc.). On two consecutive weekend days when mothers spent most of their waking hours with their child, they carried a smartphone and received prompts to complete brief surveys six times per day, every two hours between 9:00 am and 7:00 pm, yielding 12 prompts per dyad. At each prompt, mothers reported on their child’s emotion expression and their own responses to the child’s most intense negative emotion episode within the past two hours. To ensure that mothers were with their child when reporting the child’s emotion expressions, at each assessment, mothers were first asked if they were with their child. If the response was “no,” all questions regarding the child were skipped. The Ohio State University Institution Review Board approved the study procedures (Protocol #2018B0285). Mothers provided written informed consent for themselves and their children a day prior to the two-day EMA assessment, when a research assistant delivered the devices.

#### **Child positive and negative emotion expressions**

At each prompt, mothers rated their child’s momentary emotional expressions, including happy, excited, angry, anxious, sad, and stressed (e.g., “How happy was your child feeling just before the alarm went off ?”) on a 5-point scale (1 = not at all, 5 = extremely). Composite scores were computed for *positive emotion* (mean of happy and excited) and *negative emotion* (mean of angry, anxious, sad, and stressed) at each assessment. Multilevel reliability analyses indicated good reliability at both the within- and between-person levels for both positive (*α*_within_ = 0.81; *α*_between_ = 0.81) and negative emotion (*α*_within_ = 0.82, *α*_between_ = 0.81), suggesting high internal consistency across items within positive and negative emotion expressions at both within- and between-person levels (Geldhof et al., 2014).

#### Child peak negative emotion expression

At each assessment, mothers also retrospectively rated the child’s peak negative emotion (i.e., the point at which the child felt the worst) that occurred during the two-hour period after the previous assessment and prior to the current assessment (adapted from Tan et al., 2012). The intensity of child’s peak negative emotion was assessed on a 5-point scale (1 = not at all; 5 = extremely).

#### **Maternal EC and ED**

Mothers reported how they responded to the child’s peak negative emotion by selecting all applicable behaviors from a predefined list, adapted from Gottman et al. (1996): (a) ignored the child’s negative expression; (b) got upset at the child; (c) told the child to stop crying/yelling/whining; (d) told the child they were overreacting; (e) scolded or punished the child for the negative expression; (f) told the child it was okay to express emotions; (g) soothed the child or did something fun with them; (h) helped the child understand their feelings; (i) helped the child think about ways to solve the problem or improve the situation. Maternal EC and ED occurred following child peak negative emotion and prior to the current assessment.

Responses f–i were aggregated to represent *EC*, and responses a–e were aggregated to represent ED. As EC and ED items are theory-driven indicators that define the constructs, they are considered formative rather than reflective (Diamantopoulos & Winklhofer, 2001). Formative indicators are not required to be correlated, as each item contributes uniquely to the construct. Accordingly, internal consistency is not an appropriate psychometric criterion for these indices (Edwards & Bagozzi, 2000).

#### **Maternal depressive symptoms**

Before the home assessment, mothers completed the Beck Depression Inventory-II (BDI-II; Beck et al., 1996) online via Qualtrics. The BDI–II is a widely used 21-item self-report measure of current depressive symptoms rated on a 4-point scale (0–3). Internal consistency in this sample was good (*α* = 0.88). A total score was computed to represent maternal depressive symptom severity.

#### **Child negative emotionality**

Mothers completed the Child Behavioral Questionnaire (Rothbart et al., 2001), with items rated on a 7-point scale (1 = extremely untrue; 7 = extremely true). *Negative emotionality* is a second-order factor comprising subscales: anger/frustration, sadness, fear, discomfort, and falling reactivity/soothability (inversely scored), with good internal consistency (*α* = 0.88). The mean score across all items was calculated for the analyses.

### Data Analysis

Multilevel modeling (MLM) analyses were conducted using the *lmer* function from the *lme4* package in R (Bates et al., 2015). Two-level models were estimated to account for repeated assessment (level-1) nested within dyads (level-2). We first calculated the intraclass correlation (ICC) to estimate variance at the between- and within-person levels. Then, six sets of random intercept and random slope models (Model 1) were conducted to test: (1) whether child peak negative emotion was associated with maternal EC or ED (Table 2); and (2) whether maternal EC/ED predicted child’s subsequent positive and negative emotion expressions (Table 3). Lastly, we tested the extent to which maternal depressive symptoms and child negative emotionality explained variation in within-dyad associations. Maternal depressive symptoms (Model 2) and child negative emotionality (Model 3) were entered as level-2 variables in separate models. Children’s biological sex was included as a covariate in all models, given the gender differences in children’s emotion expression (Chaplin & Aldao, 2013). To disentangle within-person (level-1) and between-person (level-2) effects, level-1 predictors were group-mean centered (i.e., centered around each individual’s mean), and level-2 predictors were grand-mean centered (Hamaker & Muthén, 2020). This centering approach facilitates interpretation by isolating within-individual fluctuations from between-individual differences.

In the first two sets of MLM analyses (Table 2), the Level-1 predictor was child peak negative emotion, and the Level-2 predictor was either maternal depressive symptoms or child negative emotionality.

Level-1 model:

$$\:{Y}_{ij}=\:{\beta\:}_{0j}+\:{\beta\:}_{1j}\left({\mathrm{P}\mathrm{e}\mathrm{a}\mathrm{k}\mathrm{N}\mathrm{e}\mathrm{g}\mathrm{E}\mathrm{m}\mathrm{o}\mathrm{t}\mathrm{i}\mathrm{o}\mathrm{o}\mathrm{n}}_{ij}\right)+{\epsilon\:}_{ij}$$

Level-2 model:

$$\beta_{0j}=\gamma_{00}+\gamma_{01}\left(MaternalDepression_j\right)+\gamma_{02}(Gender_i)+\mu_{0j}$$

$$\:{\beta\:}_{1j}\:=\:{\gamma\:}_{10}\:+\:{\gamma\:}_{11}\left({\mathrm{M}\mathrm{a}\mathrm{t}\mathrm{e}\mathrm{r}\mathrm{n}\mathrm{a}\mathrm{l}\mathrm{D}\mathrm{e}\mathrm{p}\mathrm{r}\mathrm{e}\mathrm{s}\mathrm{s}\mathrm{i}\mathrm{o}\mathrm{n}}_{j}\right)+\:{\mu\:}_{1j}$$.

    

Here, $\:{Y}_{ij}$ represents maternal EC or ED for individual *j* at time *i*. When testing moderation by child negative emotionality, the same equations were used with child negative emotionality replacing maternal depression.

In the models predicting child positive or negative expressions (Table 3), maternal EC/ED occurring prior to the subsequent child emotion expressions was included as level-1 predictors. Child peak negative emotion, which elicited maternal regulatory response, was included as Level-1 covariate to account for the intensity of child negative emotion preceding mother’s regulatory attempt:

Level-1 model: $$\:\:{Y}_{ij}=\:{\beta\:}_{0j}+\:{\beta\:}_{1j}\left({\mathrm{M}\mathrm{a}\mathrm{t}\mathrm{e}\mathrm{r}\mathrm{n}\mathrm{a}\mathrm{l}\mathrm{E}\mathrm{C}}_{ij}\right){\:+\:\beta\:}_{2j}\left({\mathrm{P}\mathrm{e}\mathrm{a}\mathrm{k}\mathrm{N}\mathrm{e}\mathrm{g}\mathrm{E}\mathrm{m}\mathrm{o}\mathrm{t}\mathrm{i}\mathrm{o}\mathrm{n}}_{ij}\right)+{\epsilon\:}_{ij}$$.

Level-2 models remained the same as described above.

Across all prompts, there were 82 occasions (17.1%) in which mothers did not respond to any of the prompted questions, and 398 valid occasions on which all items were completed. On average, mothers responded to 82.9% of prompts (range = 75–100% per dyad). The Little MCAR test indicated that missingness was completely at random, *χ*^2^(9) = 0.66, *p* > .05. To reduce small-sample bias in variance component, we used restricted maximum likelihood estimation in all analyses. Further, to control the false discovery rate, we used the Benjamini–Hochberg method (Benjamini & Hochberg, 1995) to correct *p*-values (*p*_adj_) for multiple interaction tests (see Table S1 for details). For significant interaction effects after correction, we conducted Johnson–Neyman regions-of-significance tests (Figures S1- S3).

## Results

Descriptive statistics and bivariate correlations are presented in Table 1. The intensity of child peak negative emotion was positively correlated with both maternal EC (*r* = .18, *p* < .001) and ED (*r* = .23, *p* < .001). Maternal EC correlated positively with both children’s negative (*r* = .12, *p* < .05) and positive (*r* = .14, *p* < .01) emotion expressions. In contrast, maternal ED correlated with child’s higher negative expression (*r* = .16, *p* < .01) and lower positive expression (*r* = –.14, *p* < .01). Maternal EC and ED were negatively correlated (*r* = –.25, *p* < .01). ICC analyses indicated notable between-person variability for maternal EC (20%), ED (17%), and children’s positive expression (21%). Children’s negative emotion expression showed relatively less between-person variability (8%).

**Table 1:** Descriptive statistics and bivariate correlations for study variables (*N* = 40)

	Mean (SD)	Range	1	2	3	4	5	6
1. Child negative emotion expression	1.19 (0.45)	1–5	--					
2. Child positive emotion expression	3.13 (1.05)	1–5	− 0.23^***^	--				
3. Maternal emotion coaching	1.07 (0.95)	0–4	0.12^*^	0.14^**^	--			
4. Maternal emotion dismissing	0.23 (0.51)	0–2	0.16^**^	− 0.14^**^	− 0.25^***^	--		
5. Child peak negative emotion	2.48 (1.10)	1–5	0.27^***^	− 0.08	0.18^***^	0.23^***^	--	
6. Maternal depressive symptoms	9.50 (9.05)	0–35	0.18^***^	− 0.04	0.09	− 0.03	0.03	--
7. Child negative emotionality	3.58 (0.71)	0–7	0.11^*^	0.01	0.11^*^	0.09	0.11^*^	0.46^***^

^*^p<.05, ^**^p<.01, ^***^p<.001

### Child Peak Negative Emotion Predicting Maternal EC and ED

**Fig. 1:**
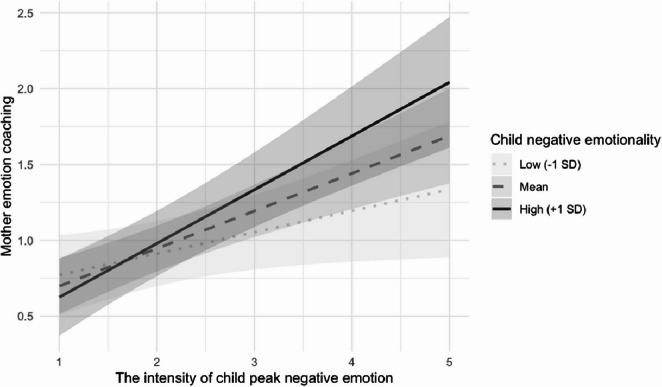
Child negative emotionality moderates the association between child peak negative emotion and maternal emotion coaching

Table 2 summarizes the results of MLMs examining the predictive associations between peak negative emotion and maternal EC and ED, and the moderating roles of maternal depressive symptoms and child negative emotionality. In Model 1, at the within-dyad level, children’s peak negative emotion was positively associated with both maternal EC (*B* = 0.26, *SE* = 0.05, *β* = 0.25, *p* < .001) and ED behaviors (*B* = 0.09, *SE* = 0.03, *β* = 0.16, *p* = .004). Further, of the cross-level interactions tested (Model 2’s and 3’s), only one was significant: child negative emotionality moderated the association between peak negative emotion and maternal EC (*B* = 0.15, *SE* = 0.07, *β* = 0.11, *p* = .023, *p*_adj_ = 0.046). As depicted in Fig. 1, child peak negative emotion predicted greater maternal EC for children with high (+ 1*SD*; *B* = 0.35, *SE* = 0.07, *p* < .001) or average negative emotionality (*B* = 0.24, *SE* = 0.05, *p* < .001), but not low negative emotionality (-1*SD*; *B* = 0.14, *SE* = 0.07, *p* > .05). The positive association between child peak negative emotion and maternal ED was not moderated by maternal depressive symptoms or child negative emotionality.

**Table 2:** Child peak negative emotion predicting maternal emotion coaching and emotion dismissing, with maternal depressive symptoms and child negative emotionality as moderators

	Maternal EC			Maternal ED		
	Model 1	Model 2	Model 3	Model 1	Model 2	Model 3
	*B (SE)*	*B (SE)*	*B (SE)*	*B (SE)*	*B (SE)*	*B (SE)*
Fixed effect						
Within-dyad						
Intercept	1.06 (0.08)^***^	1.18 (0.13)^***^	1.18 (0.12)^***^	0.23 (0.04)^***^	0.22 (0.12)	0.27 (0.11)^*^
CPNE	0.26 (0.05)^***^	0.25 (0.05)^***^	0.25 (0.05)^***^	0.09 (0.03)^**^	0.09 (0.03)^**^	0.09 (0.03)^**^
Between-dyad						
Child sex		-0.20 (0.18)	-0.21 (0.16)		0.008 (0.08)	-0.02 (0.07)
MDS		0.004 (0.01)			-0.002 (0.01)	
CPNE x MDS		0.01 (0.01)			0.0003 (0.003)	
CNE			0.12 (0.11)			0.06 (0.06)
CPNE x CNE			0.15 (0.07)^*^			0.02 (0.04)
Random effect						
Intercept	0.19 (0.44)	0.19 (0.44)	0.18 (0.43)	0.04 (0.21)	0.04 (0.21)	0.04 (0.21)
CPNE	0.03 (0.16)	0.02 (0.16)	0.02 (0.12)	0.01 (0.12)	0.02 (0.13)	0.02 (0.12)
Level-1 residual	0.65 (0.80)	0.65 (0.80)	0.65 (0.80)	0.19 (0.43)	0.19 (0.43)	0.19 (0.43)

Note.  EC=Emotion coaching; ED=Emotion dismissing; CPNE=Child peak negative emotion; CNE=Child negative emotionality; MDS=Maternal depressive symptoms.^*^p 0.05, ^**^p<.01, ^***^p < .001

### Maternal EC and ED Predicting Children’s Subsequent Emotion Expressions

Table 3 presents the MLM results testing the predictive effects of maternal EC and ED on children’s subsequent positive and negative emotion expressions, controlling for children’s prior peak negative emotion before mothers’ responses. The analysis (Model 1) revealed a positive time-lagged association between maternal EC and child positive expression (*B* = 0.16, *SE* = 0.07, *β* = 0.12, *p* = .04). This association was significant in all three models, not moderated by child negative emotionality or maternal depressive symptoms. The within-dyad association between maternal EC and subsequent child negative expression was nonsignificant (Model 1), and no moderating effect was found. However, maternal depressive symptoms were directly and positively associated with children’s negative expression (Model 2: *B* = 0.01, *SE* = 0.003, *β* = 0.25, *p* < .001).

**Table 3:** Maternal emotion coaching and dismissing predicting child positive and negative emotion expression with maternal depressive symptoms and child negative emotionality as moderators

	Child Positive Emotion Expression			Child Negative Emotion Expression		
	Maternal EC as Level-2 Predictor					
	Model 1	Model 2	Model 3	Model 1	Model 2	Model 3
	*B (SE)*	*B (SE)*	*B (SE)*	*B (SE)*	*B (SE)*	*B (SE)*
Fixed effect						
Within-dyad						
Intercept	3.14 (0.09)^***^	3.30 (0.15)^***^	3.23 (0.14)^***^	1.19 (0.03)^***^	1.13 (0.04)^***^	1.19 (0.04)^***^
Maternal EC	0.16 (0.07)^*^	0.16 (0.08)^*^	0.19 (0.07)^**^	0.01 (0.03)	0.01 (0.04)	0.01 (0.04)
CPNE	-0.11 (0.05)^*^	-0.11 (0.05)^*^	-0.11 (0.05)^*^	0.11 (0.02)^***^	0.11 (0.02)^***^	0.11 (0.02)^***^
Between-dyad						
Child sex		-0.28 (0.21)	-0.17 (0.18)		0.11 (0.06)	0.004 (0.06)
MDS		-0.01 (0.01)			0.01 (0.003)^**^	
Maternal EC x MDS		-0.0001 (0.01)			-0.004 (0.004)	
CNE			-0.006 (0.13)			0.06 (0.04)
Maternal EC x CNE ^a^			-0.21 (0.09)^*^			-0.03 (0.05)
Random effect						
Intercept	0.23 (0.48)	0.23 (0.48)	0.24 (0.49)	0.02 (0.13)	0.01 (0.10)	0.02 (0.13)
Maternal EC	0.05 (0.23)	0.06 (0.25)	0.02 (0.15)	0.02 (0.13)	0.02 (0.13)	0.02 (0.13)
Level-1 residual	0.83 (0.91)	0.83 (0.91)	0.83 (0.91)	0.16 (0.40)	0.16 (0.40)	0.16 (0.40)
Maternal ED as Level-2 Predictor						
	Model 1	Model 2	Model 3	Model 1	Model 2	Model 3
	*B (SE)*	*B (SE)*	*B (SE)*	*B (SE)*	*B (SE)*	*B (SE)*
Fixed effect						
Within-dyad						
Intercept	3.14 (0.09)^***^	2.77 (0.31)^***^	2.92 (0.28)^***^	1.19 (0.03)^***^	1.32 (0.09)^***^	1.20 (0.08)^***^
Maternal ED	-0.39 (0.11)^***^	-0.39 (0.11)^***^	-0.38 (0.11)^***^	0.14 (0.09)	0.15 (0.09)	0.11 (0.08)
CPNE	-0.05 (0.05)	-0.05 (0.05)	-0.05 (0.05)	0.10 (0.02)^***^	0.10 (0.02)^***^	0.10 (0.02)^***^
Between-dyad						
Child sex		0.26 (0.21)	0.15 (0.18)		-0.09 (0.06)	0.005 (0.06)
MDS		-0.01 (0.01)			0.01 (0.003)^***^	
Maternal ED x MDS		-0.002 (0.01)			0.02 (0.01)^*^	
CNE			0.004 (0.13)			0.06 (0.04)
Maternal ED x CNE			-0.09 (0.15)			0.33 (0.12)^**^
Random effect						
Intercept	0.23 (0.48)	0.23 (0.48)	0.24 (0.49)	0.02 (0.14)	0.01 (0.11)	0.02 (0.14)
Maternal ED	0.00 (0.03)	0.00 (0.03)	0.00 (0.02)	0.20 (0.45)	0.14 (0.37)	0.14 (0.38)
Level-1 residual	0.84 (0.92)	0.84 (0.92)	0.84 (0.92)	0.14 (0.37)	0.18 (0.43)	0.14 (0.37)

Note. EC=Emotion coaching; ED=Emotion dismissing; CPNE=Child peak negative emotion; CNE=Child negative emotionality; MDS=Maternal depressive symptom.^a^The Maternal EC x CNE effect on subsequent child positive expression was no longer significant after the Benjamini-Hochberg adjustment (*p*_adj_ = 0.07), correcting for false discovery rate, and therefore was not interpreted and probed.^*^p<.05, ^**^p<.01, ^***^p<.001

Regarding maternal ED, a within-dyad association emerged with children’s subsequent positive emotion expressions (*B* = -0.39, *SE* = 0.11, *β* = -0.16, *p* < .001), indicating that higher levels of maternal ED predicted children’s reduced positive expression, and this association was not moderated by either maternal depressive symptoms or child negative emotionality.

**Fig. 2:**
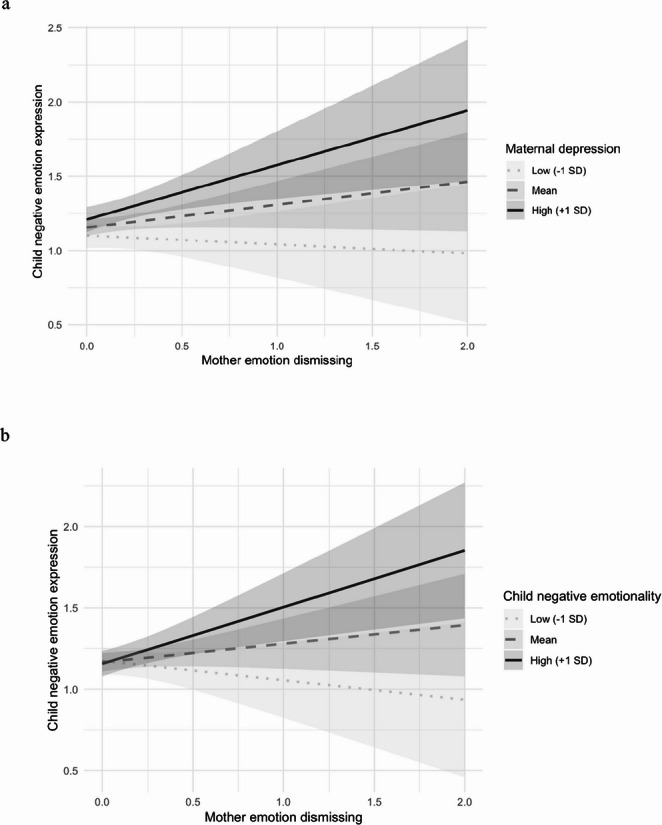
The moderating role of maternal depressive symptoms (**a**) and child negative emotionality (**b**) in the association between maternal emotion dismissing and child negative emotion expression

In contrast, the within-dyad association between maternal ED and child negative expression was initially nonsignificant. However, it varied as a function of both maternal depressive symptoms (Model 2: *B* = 0.02, *SE* = 0.01, *β* = 0.21, *p* = .019, *p*_adj_ = 0.038) and child negative emotionality (Model 3: *B* = 0.33, *SE* = 0.12, *β* = 0.23, *p* = .006, *p*_adj_ = 0.024). As shown in Fig. 2a, maternal ED predicted greater child negative emotion only among mothers with high levels of depressive symptoms (+ 1*SD*; B = 0.37, SE = 0.13, *p* = .008), but not among those with low (–1*SD*; *B* = − 0.06, *SE* = 0.13, *p* > .05) or average (*B* = 0.16, *SE* = 0.09, *p* > .05) depressive symptoms. Similarly, maternal ED predicted greater child negative emotion only for children high in negative emotionality (+ 1*SD*; *B* = 0.35, *SE* = 0.11, *p* = .005), but not for those with low (–1*SD*; *B* = − 0.12, *SE* = 0.13, *p* > .05) or average (*B* = 0.11, *SE* = 0.08, *p* > .05) negative emotionality (Fig. 2b). After the cross-level interactions were accounted for, the main effect of maternal depressive symptoms remained significant (*B* = 0.01, *SE* = 0.003, *p* < .001), indicating that mothers with higher levels of depressive symptoms had children who displayed greater negative emotion.

### Sensitivity Analyses

As the distribution of maternal ED deviated from normal (Table S2), we conducted sensitivity analyses using a dichotomized version of this variable. As shown in Tables S3 and S4, results largely remain the same (as compared to Tables 2 and 3). The only exception was that the Level-1 association between child peak negative emotion and maternal ED changed from significant to marginally significant, likely reflecting reduced power due to dichotomization.

Given the modest sample size, we also conducted post hoc Monte Carlo sensitivity analyses by simulating datasets that matched the observed data structure. We estimated the minimum detectable effect (MDE), defined as the smallest standardized coefficient yielding 80% power, and examined power across varying interaction effect sizes. Results indicated that the observed interactions between child peak negative emotion and negative emotionality, and between maternal ED and maternal depressive symptoms, were smaller than their respective MDEs, suggesting limited power to detect these effects (Figures S4 & S5). In contrast, the interaction between maternal ED and child negative emotionality was comparable to its MDE, indicating adequate power for detecting this effect (Figure S6).

## Discussion

The present study explored mother-child emotion socialization processes in naturalistic, everyday contexts. As hypothesized, we found bidirectional, time-lagged associations between children’s emotion expressions and mothers’ EC and ED behaviors. Specifically, the intensity of children’s peak negative emotion predicted mothers’ use of both EC and ED, and in turn, maternal EC/ED predicted children’s subsequent emotion expressions. However, some of these within-dyad associations were not uniform across dyads; instead, they varied as a function of maternal depressive symptoms and/or child negative emotionality.

The finding that greater intensity of children’s negative emotion predicted increased maternal ED was consistent with our hypothesis and aligned with prior research showing that children’s heightened negative emotion often evokes parental withdrawal, minimization, or punitive responses (Eisenberg et al., 1998; Pereira et al., 2022). This association tended to be robust across dyads, suggesting that ED in response to children’s intense distress may be a relatively uniform reaction among mothers.

In contrast, the positive within-dyad association between children’s peak negative emotion and maternal EC contradicted both our hypothesis and previous research, which suggests that parents typically show less supportive regulation strategies when children display high-intensity negative emotions (Del Vecchio & Rhoades, 2010; Wu et al., 2019). This association was qualified by children’s negative emotionality, such that children’s peak negative emotion predicted greater maternal EC only among children high or average (but not low) in negative emotionality. These results suggest that mothers may adjust their regulatory support in accordance with their child’s typical emotional reactivity. For children prone to intense negative emotions, mothers may engage in more active coaching to scaffold regulation, whereas for those less temperamentally reactive, mothers may allow greater autonomy in self-regulation of distress (Belsky & Pluess, 2009; Kiel et al., 2020). An alternative explanation is that the association between children’s negative emotion and maternal EC may be nonlinear. Note that the average intensity of children’s peak negative emotion in this sample was moderate (*M* = 2.48 on a 1–5 scale; Table 1), which raised the possibility that mild-to-moderate distress elicits parental empathy and increased EC, while more extreme distress may overwhelm parents and reduce their coaching behaviors. Supporting this interpretation, a laboratory study of a similar age group reported a positive time-lagged association between child negative expression and maternal comforting behavior (M. H. Chan et al., 2022).

Our data also revealed within-dyad associations between maternal regulatory behaviors and children’s subsequent emotion expressions. Supporting our hypothesis, a positive association was found between maternal EC and subsequent child positive emotion, and this association tended to be consistent across levels of maternal depressive symptoms. Previous laboratory studies have documented robust links between maternal EC and children’s positive expression and emotion regulation abilities (Ellis et al., 2014; Loop & Roskam, 2016; Lunkenheimer et al., 2007). Our findings extended this link to naturalistic, daily contexts. In contrast, maternal ED predicted reduced positive emotion in children, which is aligned with previous findings (Yap et al., 2008; Yi et al., 2016). This within-dyad association was also evident across all dyads, regardless of maternal depressive symptoms and children’s negative emotionality. This finding suggests that ED may impose immediate disruptions to children’s positive emotional experiences during mother-child interactions, even in the absence of other risk factors. The robustness of this effect also highlights the pervasive influence of ED on children’s positive emotions, potentially by limiting opportunities for shared positive emotional exchange.

In contrast to the direct and broadly negative effect on children’s positive emotion, and contrary to expectations, maternal ED did not show a within-dyad association with children’s subsequent negative expression. Rather, this association emerged only under conditions of elevated maternal depressive symptoms or high levels of child negative emotionality. These findings suggest that both maternal and child vulnerabilities may exacerbate the negative effect of maternal ED on children’s negative emotions. In line with previous research showing mixed results in the association between parental ED and child emotional outcomes, the present results indicate that the detrimental effects of parental ED on child negative emotion may only manifest under certain vulnerability conditions. This study highlighted the importance of considering moderating processes and revealed maternal depressive symptoms and child emotionality as factors that exacerbate the negative effect of maternal ED on child negative emotion.

Interestingly, while maternal ED showed a consistent association with children’s reduced positive emotion, the link between ED and negative emotions was conditioned upon child or maternal characteristics. This suggests that young children’s positive emotions may be easily disrupted by interpersonal interactions and dependent on active parental engagement. In contrast, negative emotions may be more strongly shaped by temperamental characteristics and thus require additional vulnerabilities for the effect of ED to manifest. The differential effect of maternal ED on children’s positive versus negative emotions warrants further investigation.

Finally, although both maternal depressive symptoms and child negative emotionality moderated mother-child emotional dynamics, they operated in distinct ways. While maternal depressive symptoms moderated only the effect of maternal emotion socialization behavior on children’s emotion expression, child negative emotionality moderated mother-child emotional responses in both directions. These findings highlight the importance of a transactional perspective in emphasizing children’s active role in the emotion socialization process.

### Limitations and Future Directions

Several limitations should be considered when interpreting the present findings and guiding future research. First, the sample was modest in size and consisted primarily of White, middle-class families, which limits the generalizability. Parental emotion socialization practices vary across cultural and socioeconomic contexts (Hooper et al., 2018; Parker et al., 2012). Additionally, the relative homogeneity of the sample likely reduced variability in key variables, particularly maternal depressive symptoms, which might have attenuated observed associations between maternal behaviors and child emotion expressions. Replication with larger and more diverse samples is needed to strengthen external validity of these findings. Another limitation related to the sample size concerns statistical power for detecting cross-level interaction effects. Although three interaction effects reached statistical significance, only one showed magnitude comparable to its corresponding MDE threshold, providing confidence in its robustness. The other two interactions were smaller than their MDE thresholds, suggesting that these effects were detected under conditions of limited power and should therefore be interpreted with caution. Given the exploratory nature of the present study, findings, particularly cross-level interactions, require replication in larger samples to establish their robustness. At the same time, limited power also warrants caution in interpreting the absence of significant findings.

Second, because the children were too young to self-report their emotional state, all assessments relied on maternal report. Reliance on a single informant may inflate associations between maternal behaviors and child emotion expression, raising concerns about shared method variance. Relatedly, although the temporal ordering of events was specified in the sequence of questions, all responses were reported at the same assessment point, which may limit inferences about temporal dynamics. Future studies would benefit from incorporating additional sources of information (e.g., other caregivers) or objective assessments, such as audio recordings (Gerhardt et al., 2020; Slatcher & Trentacosta, 2012).

Third, to reduce participant burden and maximize the likelihood of capturing mother–child interactions, data were collected over two weekend days. While this approach increased feasibility, it provided only a brief snapshot of daily life, and patterns of parent-child interaction during weekends may systematically differ from those during weekdays. Extending data collection to include both weekdays and weekends, or multiple bursts across several weeks, would enhance ecological validity, capture greater variability across contexts, and allow more stable estimation of within-dyad processes. Relatedly, the two-hour sampling window was selected to balance ecological validity and participant burden. However, this time window does not capture rapid transactional processes between parents and children that unfold over seconds or minutes. Future research would benefit from incorporating both short- and longer-timescale assessments to examine moment-to-moment interpersonal emotional exchanges as well as broader patterns of emotional experiences across daily life.

### Conclusion

The present study offers several notable strengths. This is among the earliest studies to use EMA with preschool-age children to capture mother–child emotion socialization processes in naturalistic home settings. This method yields ecologically valid, real-life data, advancing the field beyond retrospective reports and laboratory-based assessments. Further, this study examined emotion socialization as a bidirectional process, testing the within-dyad dynamics in mother-child mutual emotional responses, and identified important sources of heterogeneity in these transactional processes.

Our findings highlight the potential adverse effects of maternal ED behaviors on children’s everyday emotional experiences. Specifically, maternal ED predicted reduced positive emotions across all children and was particularly disadvantageous for increased child negative emotion for dyads with elevated maternal depressive symptoms or child negative emotionality. These results highlight the potential of targeting parental ED behaviors in early intervention efforts, particularly for families at heightened familial risk for depression and for children with elevated irritability or disruptive behavior, such as ODD. In addition, the consistent positive association between maternal EC and child positive emotion suggests that interventions promoting supportive parental responses may enhance children’s positive emotional experiences and help reduce dysregulation of negative emotions, potentially disrupting pathways toward ODD. Finally, the differential temporal patterns linking maternal ED with children’s positive versus negative emotions, if replicated, may inform the tailoring of intervention strategies to optimize their effectiveness.

## Supplementary Information

Below is the link to the electronic supplementary material.

Supplementary Material 1 (DOCX 505 KB)

## Data Availability

Deidentified data and materials described in this article are available from the corresponding author upon reasonable request.
