# Supplementary material for: Mother-Child Dyadic Emotional Responses in Everyday Life: Moderation by Maternal Depressive Symptoms and Child Negative Emotionality
Source: Res Child Adolesc Psychopathol. 2026 Apr 28;54(3):67. doi: 10.1007/s10802-026-01462-x (PMC13124812; doi:10.1007/s10802-026-01462-x)
Supplement: Supplementary file 1 — Supplementary Material 1 (DOCX 505 KB) [file 10802_2026_1462_MOESM1_ESM.docx]

**Supplementary Materials**

| Table S1. Benjamini–Hochberg correction for p-values by outcome variables | | | | |
| --- | --- | --- | --- | --- |
| outcome | Cross-level interaction | p-raw | Rank(i) | p-adjusted |
| Maternal EC | CPNE x CNE | 0.023 | 1 | 0.046 |
| Maternal EC | CPNE x MDS | 0.248 | 2 | 0.248 |
| Maternal ED | CPNE x CNE | 0.651 | 1 | 1.302 |
| Maternal ED | CPNE x MDS | 0.93 | 2 | 0.93 |
| Child positive | EC x CNE | 0.018 | 1 | 0.072 |
| Child positive | ED x CNE | 0.521 | 2 | 1.042 |
| Child positive | ED x MDS | 0.86 | 3 | 1.146666667 |
| Child positive | EC x MDS | 0.989 | 4 | 0.989 |
| Child negative | ED x CNE | 0.006 | 1 | 0.024 |
| Child negative | ED x MDS | 0.019 | 2 | 0.038 |
| Child negative | EC x MDS | 0.254 | 3 | 0.338666667 |
| Child negative | EC x CNE | 0.474 | 4 | 0.474 |

EC=Emotion coaching; ED=Emotion dismissing; CPNE=Child peak negative emotion; CNE=Child negative emotionality; MDS=Maternal depressive symptom.

We applied the Benjamini–Hochberg adjustment, a widely used method for controlling the false discovery rate (Benjamini & Hochberg, 1995), to adjust the p-values of the interaction effects. Following Rubin’s (2017) recommendation, we defined the “family” of tests as the number of interaction effects examined for each dependent variable. The adjustment was therefore conducted separately for the set of interactions predicting each child outcome.

References:

Benjamini, Y., & Hochberg, Y. (1995). *Controlling the false discovery rate: A practical and powerful approach to multiple testing*. *Journal of the Royal Statistical Society: Series B (Methodological), 57*(1), 289–300. <https://doi.org/10.1111/j.2517-6161.1995.tb02031.x>

Rubin, M. (2017). *Do p values lose their meaning in exploratory analyses? It depends how you define the familywise error rate*. *Review of General Psychology, 21*(2), 269–275. <https://doi.org/10.1037/gpr0000097>

**Sensitivity analyses**

***Using dichotomized maternal ED in the analyses.*** As the distribution of maternal ED deviated from normal (see distribution in Table S2 below), we ran the sensitivity analysis after dichotomizing this variable. Results are shown in Table S3 and S4.

Table S2 Frequency distribution of maternal ED

| Emotion dismissing | Frequency | Percentage |
| --- | --- | --- |
| 0 | 319 | 80% |
| 1 | 66 | 17% |
| 2 | 13 | 3% |

Table S3. Child peak negative emotion predicting maternal emotion dismissing (dichotomized), with maternal depressive symptoms and child negative emotionality as moderators

|  | Maternal ED | | |
| --- | --- | --- | --- |
|  | Model 1 | Model 2 | Model 3 |
|  | *B (SE)* | *B (SE)* | *B (SE)* |
| Fixed effect |  |  |  |
| Within-individual |  |  |  |
| Intercept | -1.68 (.24)^***^ | -1.56 (.76)^*^ | -1.32 (.67)* |
| CPNE | 0.34 (.19)^+^ | 0.34 (.19)^+^ | 0.33 (.19)^+^ |
| Between-individual |  |  |  |
| Child sex |  | -0.09 (.51) | -0.26 (.45) |
| MDS |  | -0.007 (.03) |  |
| CPNE x MDS |  | 0.01 (.02) |  |
| CNE |  |  | 0.45 (.31) |
| CPNE x CNE |  |  | 0.03 (.22) |
|  |  |  |  |
| Random effect |  |  |  |
| Intercept | 0.99 (.15) | 0.99 (.15) | 0.95 (.23) |
| CPNE | 0.03 (.02) | 0.03 (.49) | 0.04 (.57) |
| Level-1 residual | - | - | - |

^+^*p*<.10, ^*^*p*<.05, ^**^*p*<.01, ^***^*p*<.001

Table S4. Maternal emotion dismissing (dichotomized) predicting child positive and negative emotion expression with maternal depressive symptoms and child negative emotionality as moderators

|  | Positive Emotion | | | Negative Emotion | | |
| --- | --- | --- | --- | --- | --- | --- |
|  | Model 1 | Model 2 | Model 3 | Model 1 | Model 2 | Model 3 |
|  | *B (SE)* | *B (SE)* | *B (SE)* | *B (SE)* | *B (SE)* | *B (SE)* |
| Fixed effect |  |  |  |  |  |  |
| Within-individual |  |  |  |  |  |  |
| Intercept | 3.20 (.09)^***^ | 2.84 (.32)^***^ | 3.00 (.28)^***^ | 1.17 (.03)^***^ | 1.26 (.09)^***^ | 1.14 (.09)^***^ |
| Maternal ED | -0.34 (.13)^**^ | -0.34 (.13)^**^ | -0.34 (.13)^*^ | 0.14 (.10) | 0.15 (.10) | 0.10 (.09) |
| CPNE | -0.07 (.05) | -0.07 (.05) | -0.07 (.05) | 0.11 (.02)^***^ | 0.11 (.02)^***^ | 0.11 (.02)^***^ |
| Between-individual |  |  |  |  |  |  |
| Child sex |  | 0.26 (.21) | 0.15 (.19) |  | -0.06 (.06) | 0.03 (.06) |
| MDS |  | -0.01 (.01) |  |  | 0.009 (.003)^**^ |  |
| Maternal ED x MDS |  | -0.007 (.01) |  |  | 0.02 (.01)^*^ |  |
| CNE |  |  | 0.02 (.13) |  |  | 0.02 (.04) |
| Maternal ED x CNE |  |  | -0.01 (.19) |  |  | 0.33 (.13)^*^ |
|  |  |  |  |  |  |  |
| Random effect |  |  |  |  |  |  |
| Intercept | 0.23 (.48) | 0.23 (.48) | 0.24 (.49) | 0.01 (.11) | 0.01 (.10) | 0.01 (.12) |
| Maternal ED | 0.00 (.03) | 0.00 (.02) | 0.00 (.03) | 0.20 (.45) | 0.19 (.43) | 0.16 (.40) |
| Level-1 residual | 0.85 (.92) | 0.86 (.93) | 0.86 (.93) | 0.15 (.38) | 0.15 (.38) | 0.15 (.38) |
| Note. EC=Emotion coaching; ED=Emotion dismissing; CPNE=Child peak negative emotion; CNE=Child negative emotionality; MDS=Maternal depressive symptom.  ^*^*p*<.05, ^**^*p*<.01, ^***^*p*<.001 | | | | | | |


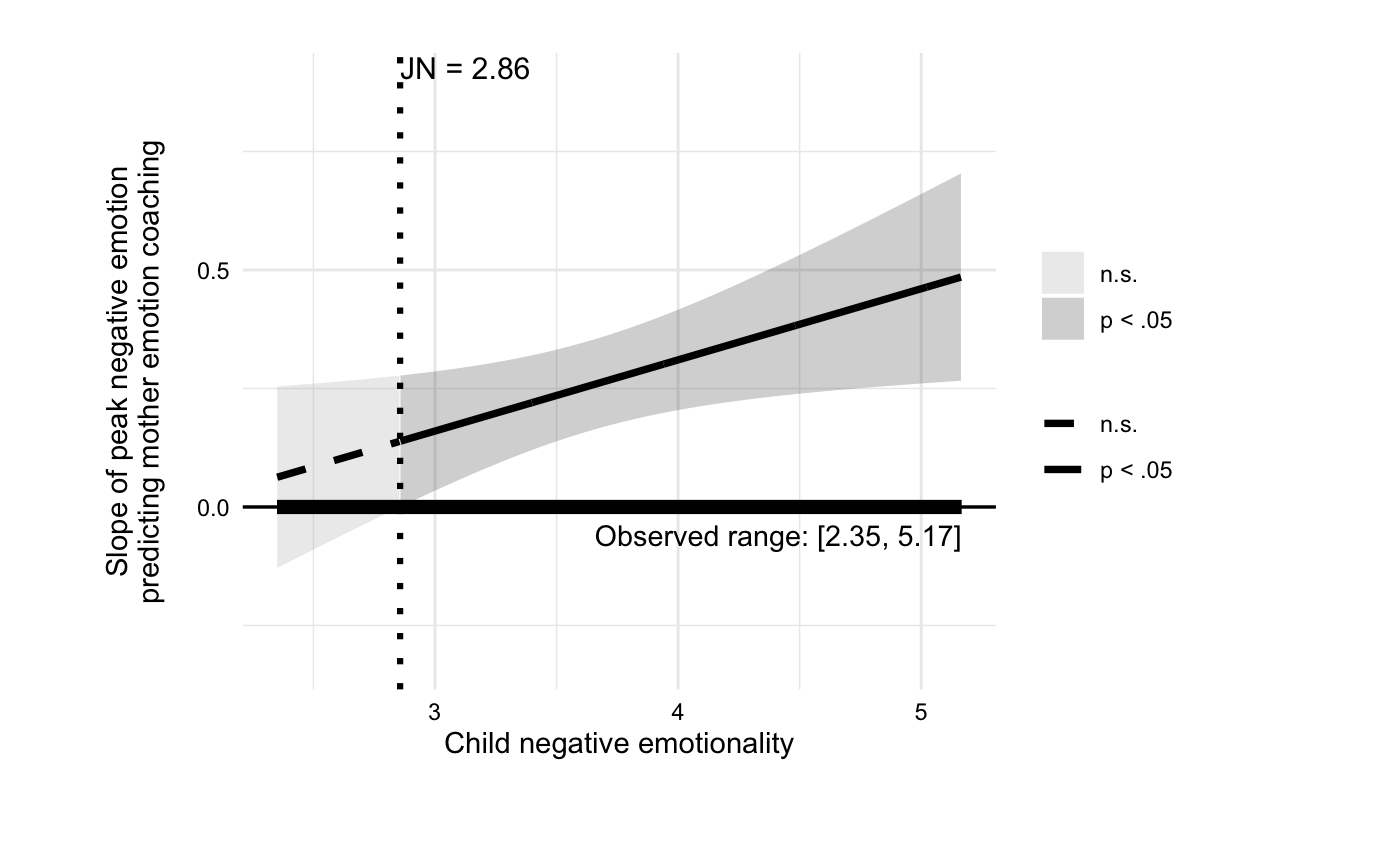


Figure S1. Johnson–Neyman regions-of-significance plot showing the slope of peak negative emotion predicting maternal emotion coaching across levels of child negative emotionality.

As shown in Figure S1, the association between peak negative emotion and maternal EC became statistically significant when child negative emotionality exceeded 2.86 on the original scale. Importantly, this region lies within the observed range of the moderator in our sample. Approximately 77.5% of children (31 out of 40) had levels of negative emotionality above this threshold, indicating that the moderating effect applies to a substantial proportion of the sample.


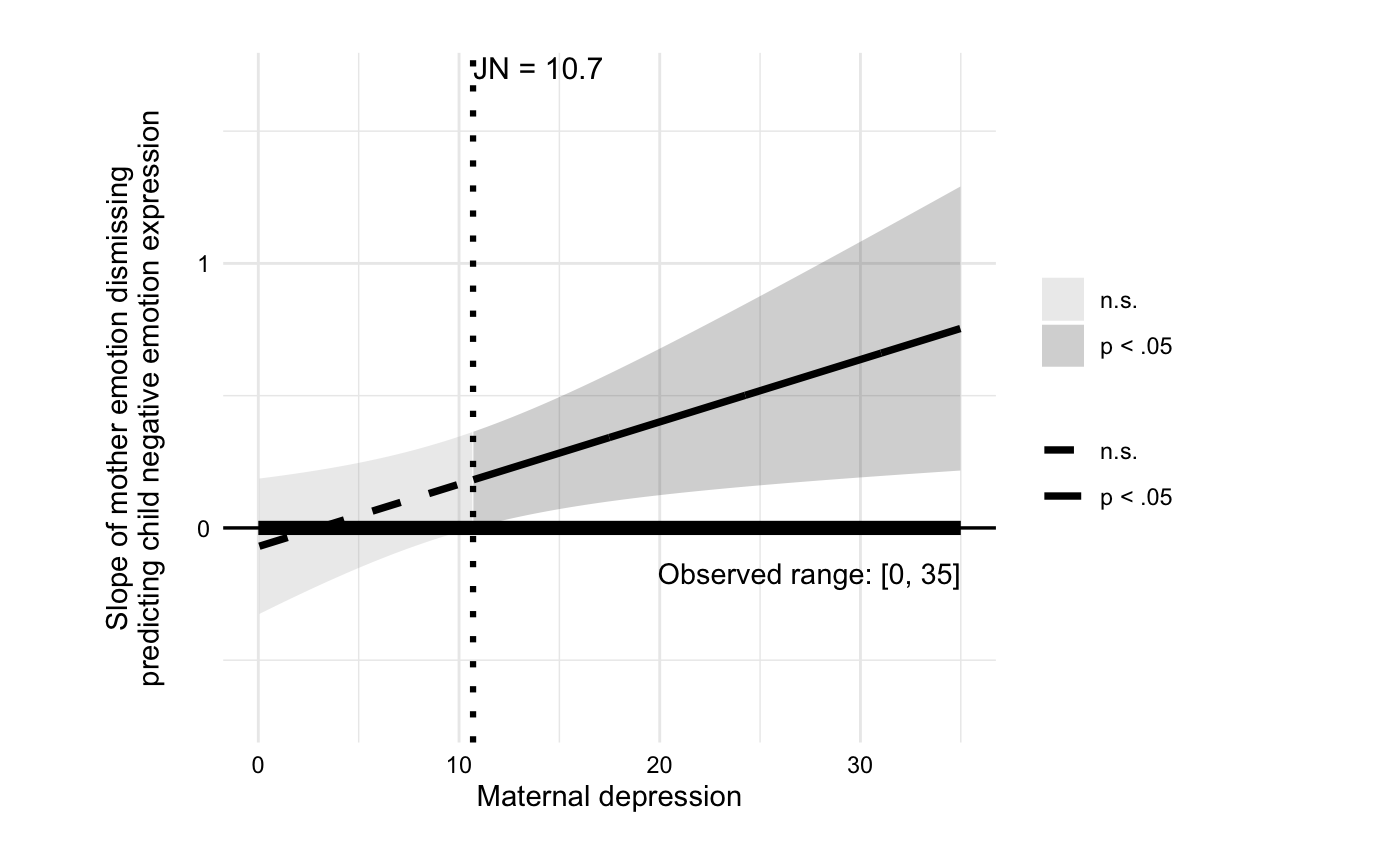


Figure S2. Johnson–Neyman regions-of-significance plot showing the slope of maternal emotion dismissing predicting child negative emotion expression across levels of maternal depression.

The Johnson–Neyman regions-of-significance tests indicated that the association became statistically significant when maternal depressive symptoms exceeded 10.70, and approximately 35% of mothers (14 out of 40) had depressive symptoms above this threshold.


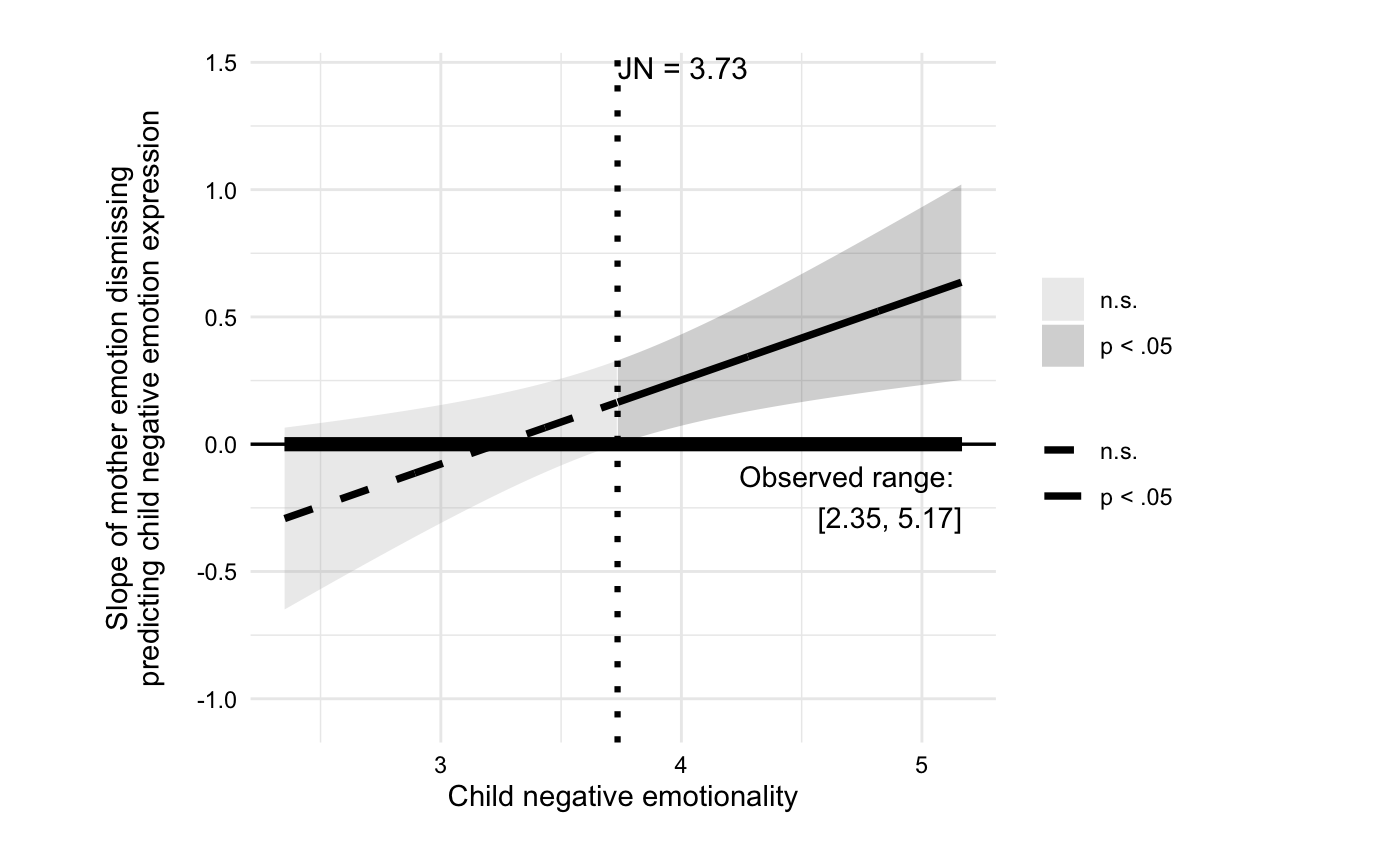


Figure S3. Johnson–Neyman regions-of-significance plot showing the slope of mother emotion dismissing predicting child negative emotion expression across levels of child negative emotionality.

As shown in Figure S3, the association became statistically significant when child negative emotionality exceeded 3.73, and 40% of children (16 out of 40) had levels of child negative emotionality above this threshold. Thus, the Johnson–Neyman regions-of-significance tests indicated that these moderating effects apply to a meaningful proportion of the sample.


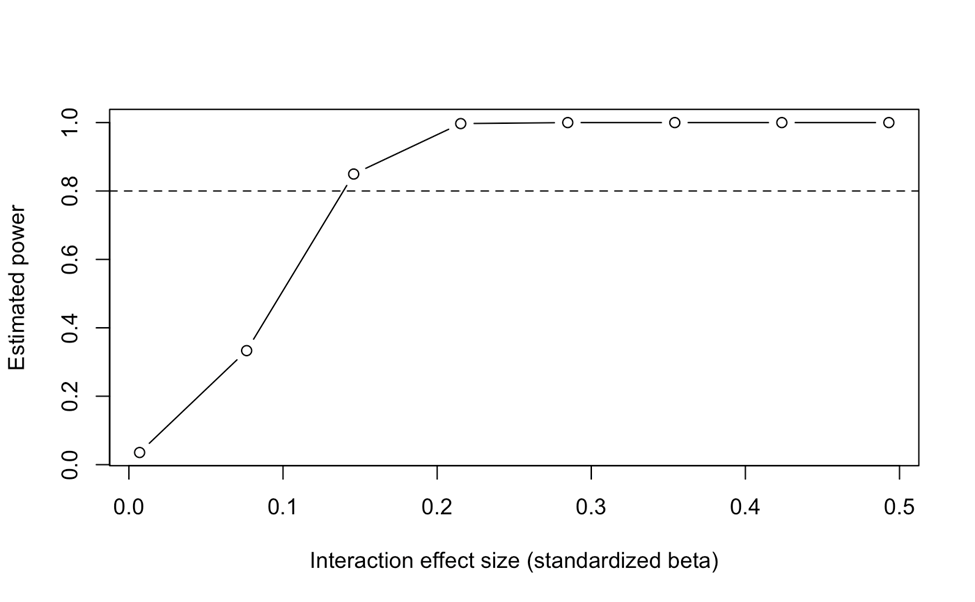


Figure S4. Power and minimum detectable effect size for the interaction between child peak negative emotion intensity and negative emotionality in predicting maternal emotion coaching. The observed effect (unstandardized B = 0.15) was smaller than the detectable threshold (unstandardized MDE = 0.20; standardized MDE = 0.14), suggesting limited power for detecting effects of this magnitude.


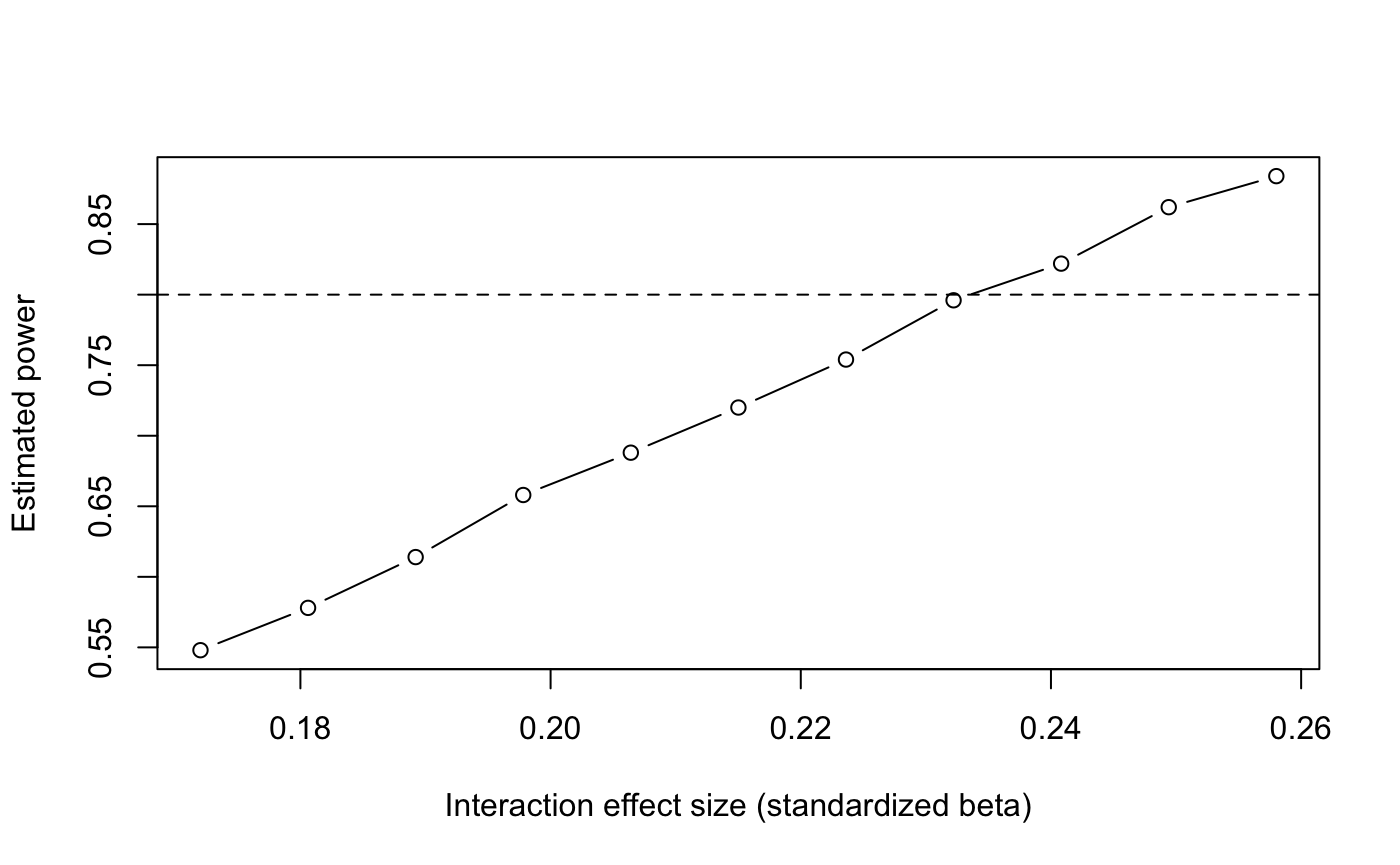


Figure S5. Power and minimum detectable effect for the interaction between maternal ED and maternal depressive symptoms in predicting child negative emotion expression. The observed effect (unstandardized B = 0.02) was smaller than the detectable threshold (unstandardized MDE = 0.03; standardized MDE = 0.24), suggesting limited power and a higher likelihood of Type II error.


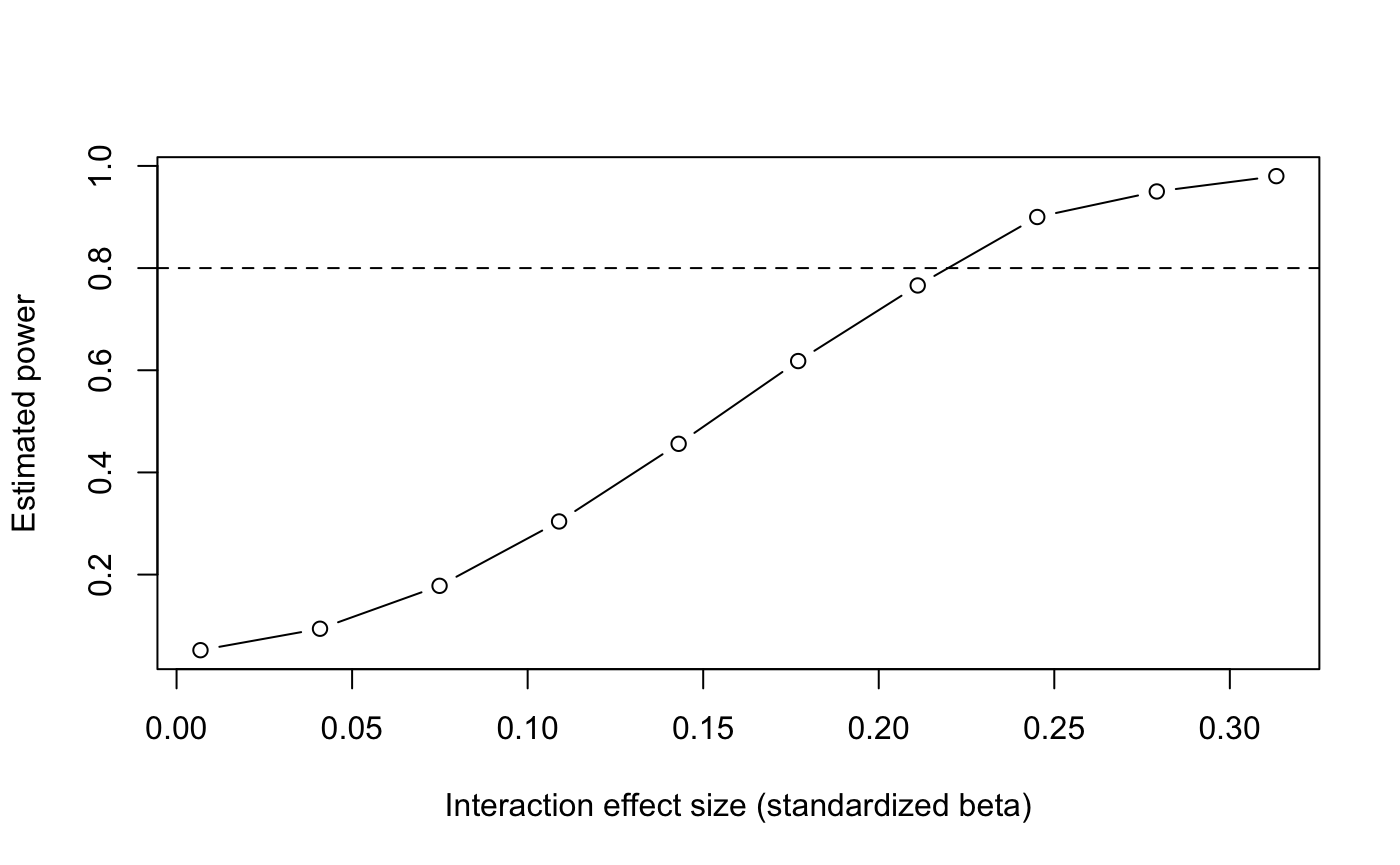


Figure S6. Power and minimum detectable effect for the interaction between maternal ED and child negative emotionality in predicting child negative emotion expression. The observed effect (unstandardized B = 0.33) was comparable to the detectable threshold (unstandardized MDE = 0.33; standardized MDE = 0.22), indicating adequate power for detecting this interaction effect.
